# Supplementary material for: The PREDEP-SERT study protocol: A 6-month follow-up cohort study of predictors of effectiveness, tolerability and safety of sertraline for depression using Therapeutic Drug Monitoring
Source: PLoS One. 2025 Aug 8;20(8):e0325335. doi: 10.1371/journal.pone.0325335 (PMC12333983; doi:10.1371/journal.pone.0325335)
Supplement: S1 File — (DOC) [file pone.0325335.s001.doc]

# PROTOCOLO

**1. TÍTULO DESCRIPTIVO Y VERSIÓN DEL PROTOCOLO.**

Estudio de predictores de eficacia y tolerabilidad del tratamiento antidepresivo en pacientes con depresión mayor tratados con sertralina

Versión 3, de fecha 6 de marzo de 2024

**2. PROMOTOR DEL ESTUDIO:**

CLINICA UNIVERSIDAD DE NAVARRA

Avenida Pío XII, 36

CP: 31008 Pamplona

**3. RESPONSABLES DEL ESTUDIO.**

Azucena Aldaz Pastor. Doctora en Farmacia. Especialista en Farmacia Hospitalaria. Clínica Universidad de Navarra.

Felipe Ortuño Sánchez Pedreño. Doctor en Medicina. Especialista en Psiquiatría. Clínica Universidad de Navarra.

Begoña Tapia Alzuguren. Graduada en Farmacia. Farmacéutica Interna Residente (Farmacia Hospitalaria). Clínica Universidad de Navarra.

Covandonga Canga Espina. Graduada en Medicina. Médica Interna Residente (Psiquiatría). Clínica Universidad de Navarra.

María del Mar Unceta González. Licenciada en Medicina. Especialista en Psiquiatría. Clínica Universidad de Navarra.

Enrique Aubá Guedea. Doctor en Medicina. Especialista en Psiquiatría. Clínica Universidad de Navarra.

José Pablo Bullard García Naranjo. Licenciado en Medicina. Especialista en Psiquiatría. Clínica Universidad de Navarra.

Coordinador: Patricio Molero Santos. Doctor en Medicina. Especialista en Psiquiatría. Clínica Universidad de Navarra.

**4. RESUMEN.**

a) Título y subtítulos, con la versión y la fecha del protocolo, nombre y apellido del autor principal y organismo para el que trabaja.

Título: Estudio de predictores de eficacia y tolerabilidad del tratamiento antidepresivo en pacientes con depresión mayor tratados con sertralina

Título corto: Predictores de respuesta en depresión-SERT (PREDEP-SERT)

Versión 3, de fecha 06 de marzo de 2024

Co-IP: Azucena Aldaz y Patricio Molero. Clínica Universidad de Navarra

b) Justificación y contexto.

El trastorno depresivo mayor (TDM) es una enfermedad grave, recurrente e incapacitante con una elevada prevalencia en nuestro medio, que se asocia a un incremento de morbimortalidad por riesgo de suicidio y otras causas médicas y psiquiátricas asociadas, y supone una de las causas más importantes de años de vida perdidos ajustados por discapacidad. La farmacoterapia antidepresiva es una parte fundamental del tratamiento del TDM, y como primera línea se recomienda un inhibidor selectivo de la recaptación de serotonina (ISRS). Durante las primeras semanas de tratamiento se realiza un ajuste de dosis para identificar la dosis mínima eficaz (DME) del paciente, que combine eficacia y aceptable tolerabilidad. El ajuste de dosis del ISRS se realiza según la ficha técnica y el juicio clínico. Determinados condicionantes genéticos y ambientales pueden facilitar la aparición de toxicidad o falta de eficacia a los rangos de dosis establecidos, con necesidad de utilizar dosis menores o mayores, y riesgo de alargamiento excesivo de la latencia de respuesta que puede asociarse a complicaciones. Estas situaciones suelen manejarse según juicio clínico de forma individual, no hay un consenso establecido en guías clínicas que permita ajustar las dosis de manera personalizada, y la determinación de niveles plasmáticos puede ser de utilidad en este ámbito. Un ejemplo de ISRS para el que existe evidencia preliminar de la importancia de ajuste de dosis en función de sus niveles plasmáticos es la sertralina, con rangos óptimos de concentraciones plasmáticas bajas o intermedias asociados a mayor eficacia, de modo que incrementos a partir de determinadas dosis, aun permitidas por ficha técnica, no aportarían ningún beneficio clínico. Es necesaria una mayor evidencia del rango terapéutico óptimo de este fármaco en términos de eficacia y tolerabilidad. A través de este estudio se pretende investigar si existen unos rangos de concentración plasmática de sertralina óptimos asociados a la respuesta terapéutica y a una aceptable tolerabilidad a partir de la segunda semana y en los primeros 6 meses de tratamiento, y cuáles son los determinantes sintomatológicos, fisiopatológicos, genéticos y ambientales de utilidad clínica que pueden modificarlos.

c) Hipótesis y objetivos de la investigación.

Hipótesis de trabajo:

Existe una asociación entre la concentración plasmática de sertralina y el grado de intensidad de los síntomas depresivos a los 6 meses de tratamiento.

Objetivo primario:

Estudiar la correlación entre la concentración plasmática de sertralina y la intensidad de los síntomas depresivos a través de la escala de Hamilton para la Depresión de 21 ítems a los 6 meses de tratamiento.

Objetivos secundarios:

Valorar la respuesta clínica global al tratamiento íntegro, tanto farmacológico como no farmacológico, e identificar los factores determinantes genéticos y ambientales de la respuesta al tratamiento en pacientes diagnosticados de TDM de acuerdo con las siguientes variables:

1. Estudiar la asociación entre la concentración plasmática de sertralina y la respuesta (disminución del 50% de la intensidad de los síntomas depresivos a través de la escala de Hamilton para la Depresión de 21 ítems) y remisión (puntuación en dicha de escala menor o igual a 7) de los síntomas depresivos a los 6 meses de tratamiento.

2. Estudiar la correlación entre la mejoría del perfil sintomático global y la tolerabilidad y las concentraciones plasmáticas de sertralina a los 15 días, 30 días, 60 días, 90 días y 6 meses de los pacientes con TDM evaluado a través de las siguientes escalas: escala de Hamilton para la Depresión de 21 ítems, Montgomery-Asberg para la depresión, Hamilton para la Ansiedad, Impresión Clínica Global, Inventario de Depresión de Beck, escala Columbia para el riesgo de suicidio, Yale-Brown para síntomas obsesivo-compulsivos, valoración cognitiva (exclusivamente basal y a los 6 meses) y listado de efectos adversos.

3. Describir la utilidad del uso conjunto de marcadores farmacogenéticos relacionados con la respuesta antidepresiva (principalmente isoformas CYP2B6 y CYP2C19) y la monitorización farmacocinética en la optimización farmacológica del tratamiento del TDM de esta muestra de pacientes.

4. Describir la influencia de antecedentes familiares, hábitos tóxicos, estilos de vida, condicionantes sociales, laborales y familiares en la evolución del TDM de esta muestra de pacientes.

5. Determinar la implicación de las medidas no farmacológicas en la evolución del TDM de esta muestra de pacientes (medidas higiénico-dietéticas de estilo de vida, psicoterapia, terapia electroconvulsiva).

d) Diseño del estudio.

Observacional, prospectivo y retrospectivo.

e) Población.

Pacientes con sintomatología depresiva primaria o secundaria en tratamiento farmacológico antidepresivo con sertralina del departamento de Psiquiatría de la Clínica Universidad de Navarra, con los siguientes criterios de inclusión y sin el criterio de exclusión:

Criterios de inclusión/exclusión

Inclusión

Pacientes de cualquier edad en tratamiento con sertralina por presentar sintomatología depresiva primaria o secundaria a otros diagnósticos psiquiátricos o médicos Exclusión

Sospecha de falta de adherencia al tratamiento.

f) Variables.

-Variables antropométricas, demográficas y hábitos de vida (basal, antes del inicio de tratamiento): Sexo, edad, estado civil, lugar en la fratria, talla, peso, IMC, superficie corporal, práctica de ejercicio físico (intensidad, frecuencia), dieta (saludable/no saludable), grado de formación escolar, profesional o académica, actividad de cuidado familiar (cuidado de hijos o familiares dependientes), actividad laboral (sí/no), creencias/prácticas religiosas (sí/no), relaciones sociales (mantenida/empobrecida/nula-soledad), residencia (urbana/rural).

-Diagnóstico principal (basal)

-Comorbilidades psiquiátricas y médicas generales (basal)

-Variables de tratamiento farmacológico (basal, a los 15 (+/-2) días, 30 (+/-5) días, 60(+/-10) días, 90(+/-15) días y 180 (+/-15) días): Dosis, intervalo de dosificación, hora de administración, duración de tratamiento con esa dosis, comedicación incluyendo su posología y duración. En la rama prospectiva se realizará una triple confirmación de cumplimiento de adherencia: prescripción médica, referida verbalmente por el paciente, referida verbalmente por acompañante del paciente (cuando sea posible y con previa autorización del paciente).

-Variables farmacocinéticas(basal, a los 15 (+/-2) días, 30 (+/-5) días, 60(+/-10) días, 90(+/-15) días y 180 (+/-15) días) : Fecha de extracción, hora de extracción, concentraciones séricas de sertralina y n-desmetilsertralina.

-Variables de eficacia (basal, a los 15 (+/-2) días, 30 (+/-5) días, 60(+/-10) días, 90(+/-15) días y 180 (+/-15) días) : a través de las siguientes escalas de intensidad sintomática: Hamilton para la Depresión, Montgomery-Asberg para la depresión, Hamilton para la Ansiedad, Impresión Clínica Global, Inventario de Depresión de Beck, escala Columbia para el riesgo de suicidio, Yale-Brown para síntomas obsesivo-compulsivos, valoración cognitiva (exclusivamente basal y a los 6 meses).

-Variables de tolerabilidad (basal, a los 15 (+/-2) días, 30 (+/-5) días, 60(+/-10) días, 90(+/-15) días y 180 (+/-15) días) : recogida de efectos adversos: listado y graduación.

-Variables de tratamiento no farmacológico (basal, a los 15 (+/-2) días, 30 (+/-5) días, 60(+/-10) días, 90(+/-15) días y 180 (+/-15) días) : Medidas higiénico-dietéticas de estilo de vida (higiene del sueño, patrón de dieta saludable, ejercicio físico regular, abandono de tabaco y/o tóxicos). Tipo de psicoterapia, Cantidad/frecuencia de sesiones de psicoterapia, terapia electroconvulsiva (tipo y frecuencia).

-Variables analíticas (al inicio de tratamiento y posteriormente hasta los 180 (+/-15)días, si existen): Hemograma, ionograma, Vit. B1, B6, B12, folato, ferritina, albumina plasmática, ALT, AST, bilirrubina total y directa, fosfatasa alcalina, urea y creatinina.

-Variables genéticas (si se cuenta con análisis farmacogenético por criterio clínico): isoformas CYP, principalmente isoformas CYP2B6 y CYP2C19

-Conducta o ideación suicida (a lo largo de la vida,basal, a los 15 (+/-2) días, 30 (+/-5) días, 60(+/-10) días, 90(+/-15) días y 180 (+/-15) días)

-Hábitos tóxicos (a lo largo de la vida,basal, a los 15 (+/-2) días, 30 (+/-5) días, 60(+/-10) días, 90(+/-15) días y 180 (+/-15) días): consumo de alcohol, tabaco u otros tóxicos, adicciones conductuales

-Otras variables (basal, a los 15 (+/-2) días, 30 (+/-5) días, 60(+/-10) días, 90(+/-15) días y 180 (+/-15) días): antecedentes psiquiátricos familiares, acontecimientos vitales estresantes-precipitantes/traumáticos (también a lo largo de la vida), situación familiar de dependencia (también a lo largo de la vida), expectativas sobre el tratamiento farmacológico, conciencia de enfermedad.

g) Fuentes de los datos.

Todas las variables analizadas se recogen en la historia psiquiátrica por práctica habitual. Los datos se obtendrán mediante la revisión de historias clínicas en el sistema electrónico de historias clínicas de la CUN.

h) Tamaño de muestra del estudio.

Se ha estimado un tamaño de muestra necesario para realizar el estudio de al menos 61 pacientes para alcanzar una potencia estadística del 80% para detectar diferencias considerando un coeficiente de correlación de 0,35 frente a una hipótesis nula de no correlación, considerando un test de hipótesis a dos colas y un nivel de significación del 5%.

i) Análisis de los datos.

Se realizará un análisis descriptivo incluyendo el cálculo de la media y la desviación estándar para variables cuantitativas, y de porcentajes para variables cualitativas. Se empleará el coeficiente de correlación de Pearson para valorar la asociación entre la concentración plasmática de sertralina y el grado de intensidad de los síntomas depresivos. Se calculará la odds ratio y su respectivo intervalo de confianza al 95% como medida de asociación utilizando modelos de regresión logística. Los valores p<0.05 serán considerados para establecer la significación estadística. Los análisis estadísticos serán realizados con el programa Stata 14 (StataCorp. 2015. Stata Statistical Software: Release 14. College Station, TX: StataCorp LP).

j) Etapas y calendario.

| **Actividad** | **Fecha inicio** | **Fecha fin** |
| --- | --- | --- |
| Recogida de datos | Julio 2021 | Diciembre 2026 |
| Análisis e interpretación de los datos obtenidos | enero 2022 | Febrero 2027 |
| Redacción | Marzo 2022 | Junio 2027 |
| Informe final de resultados | Septiembre 2027 |  |

**5. MODIFICACIONES Y ACTUALIZACIONES**

Toda modificación sustancial del protocolo del estudio después del inicio de la recogida de los datos, con su justificación, la fecha y la indicación de la sección del protocolo que se ha visto afectada.

**6. ETAPAS**

Cuadro con el calendario previsto para, al menos, las siguientes etapas:

a) Inicio de la recogida de datos.

b) Final de la recogida de datos.

c) Informes de situación del estudio, si procede. (No procede).

d) Informes intermedios de los resultados del estudio, si procede. (No procede).

e) Informe final de los resultados del estudio.

| **Actividad** | **Fecha inicio** | **Fecha fin** |
| --- | --- | --- |
| Recogida de datos | Julio 2021 | Diciembre 2026 |
| Análisis e interpretación de los datos obtenidos | enero 2022 | Febrero 2027 |
| Redacción | Marzo 2022 | Junio 2027 |
| Informe final de resultados | Septiembre 2027 |  |

**7. JUSTIFICACIÓN Y CONTEXTO.**

El trastorno depresivo mayor (TDM) según la DSM-5 (categorías de episodios depresivos moderados o graves según la CIE-10) es una enfermedad grave, recurrente e incapacitante con una elevada prevalencia en nuestro medio (con estimaciones de la prevalencia-vida media de un 14,6% (3), y prevalencia global en Europa de un 8,56% (4)), que ocasiona un deterioro de la salud médica general, el funcionamiento sociofamiliar, el rendimiento laboral y la calidad de vida. Se asocia a un incremento de morbimortalidad por riesgo de suicidio y otras causas médicas y psiquiátricas asociadas (5). Supone una de las causas mundiales más importantes (la tercera-cuarta en nuestro entorno) de años de vida perdidos ajustados por discapacidad (DALYs), y se estima que será la primera causa de DALYs a nivel mundial en 2030 (6).

El tratamiento del TDM consiste en una combinación de farmacoterapia antidepresiva y psicoterapia, y terapia electroconvulsiva en casos de riesgo vital, gravedad y/o refractariedad que requieran respuesta rápida (7). La farmacoterapia antidepresiva es una parte fundamental del manejo del TDM, y como primera línea de tratamiento se recomienda un inhibidor selectivo de la recaptación de serotonina (ISRS) (7), grupo farmacológico ampliamente utilizado para tratar el TDM, relativamente seguro pero con el inconveniente de una latencia de respuesta prolongada hasta el inicio de la mejoría clínica significativa (hasta 5-8 semanas) (8). Durante las primeras semanas de tratamiento se realiza un ajuste de dosis para identificar la dosis mínima eficaz (DME) del paciente, que combine eficacia (respuesta antidepresiva: reducción de al menos un 50% de la intensidad de los síntomas previos al tratamiento en escalas específicas (9)) y aceptable tolerabilidad. Dosis inferiores a la DME se asocian a riesgo de complicaciones del TDM por empeoramiento depresivo, mientras que dosis superiores pueden asociarse a un mayor riesgo de efectos adversos que a su vez condicione una disminución de la adherencia al tratamiento (10), un fenómeno que suele ocurrir en las primeras semanas y se asocia a mayor riesgo de recaída o recurrencia depresiva (11). Actualmente, el ajuste de dosis del ISRS se realiza según la ficha técnica y el juicio clínico. Sin embargo, hay evidencia creciente de determinados condicionantes genéticos que pueden facilitar la aparición de toxicidad o falta de eficacia a los rangos de dosis establecidos en algunas situaciones, con necesidad de utilizar dosis menores o mayores (12,13). Estas situaciones suelen manejarse según juicio clínico de forma individual (excepcionalmente con guía farmacogenética), y no hay un consenso establecido en guías clínicas que permita ajustar las dosis de manera personalizada. Aunque la determinación de niveles plasmáticos de antidepresivos puede ser muy útil en el manejo de algunos casos no respondedores o con toxicidad, actualmente no se conocen los rangos de concentraciones plasmáticas óptimas. Un ejemplo de ISRS para el que existe evidencia preliminar de la importancia de ajuste de dosis en función de sus niveles plasmáticos es la sertralina (1,2,14), con resultados dispares: por una parte se ha establecido un consenso de un rango terapéutico amplio (10-150ng/ml) (14), aunque hay evidencia preliminar de una relación curvilínea entre mejoría clínica y concentración plasmática, con mayor eficacia en rangos de concentraciones plasmáticas bajas o intermedias (25-50ng/ml o 40-70ng/ml) (1,2), de modo que incrementos a partir de determinadas dosis, aun permitidas por ficha técnica, no aportarían ningún beneficio clínico. Aclarar este aspecto es importante clínicamente, ya que los incrementos de dosis van ligados a alargamientos de una latencia de respuesta ya de por sí prolongada en los ISRS, que pueden asociarse a empeoramientos o complicaciones del TDM por una pérdida de oportunidad de un cambio precoz a un tratamiento más eficaz y mejor tolerado (potenciación o relevo farmacológicos). Por otra parte, determinados polimorfismos genéticos de los grupos enzimáticos implicados en el metabolismo hepático de primer paso de la sertralina (CYP3A4, CYP2C19 y CYP2B6) pueden condicionar sus concentraciones plasmáticas, y esta actividad enzimática puede modificarse por determinadas interacciones farmacológicas. Por todo ello, es necesaria una mayor evidencia del rango terapéutico óptimo de la sertralina en términos de eficacia y tolerabilidad, así como del papel de la farmacocinética como elemento modulador clave de la relación entre los marcadores farmacogenéticos relacionados con este fármaco y su eficacia y tolerabilidad.

A través de este estudio se pretende investigar si existen unos rangos de concentración plasmática de sertralina óptimos asociados a la respuesta terapéutica y a una aceptable tolerabilidad a partir de la segunda semana y en los primeros 6 meses de tratamiento, y cuáles son los determinantes sintomatológicos, fisiopatológicos, genéticos y ambientales de utilidad clínica que pueden modificarlos.

**8. HIPÓTESIS Y OBJETIVOS DE LA INVESTIGACIÓN**

**HIPÓTESIS**

Existe una asociación entre la concentración plasmática de sertralina y el grado de intensidad de los síntomas depresivos a los 6 meses de tratamiento.

**OBJETIVOS PRIMARIOS Y SECUNDARIOS.**

Objetivo primario:

Estudiar la correlación entre la concentración plasmática de sertralina y la intensidad de los síntomas depresivos a través de la escala de Hamilton para la Depresión de 21 ítems a los 6 meses de tratamiento.

Objetivos secundarios:

Valorar la respuesta clínica global al tratamiento íntegro, tanto farmacológico como no farmacológico, e identificar los factores determinantes genéticos y ambientales de la respuesta al tratamiento en pacientes diagnosticados de TDM de acuerdo con las siguientes variables:

1. Estudiar la asociación entre la concentración plasmática de sertralina y la respuesta (disminución del 50% de la intensidad de los síntomas depresivos a través de la escala de Hamilton para la Depresión de 21 ítems) y remisión (puntuación en dicha de escala menor o igual a 7) de los síntomas depresivos a los 6 meses de tratamiento.

2. Estudiar la correlación entre la mejoría del perfil sintomático global y la tolerabilidad y las concentraciones plasmáticas de sertralina a los 15 días, 30 días, 60 días, 90 días y 6 meses de los pacientes con TDM evaluado a través de las siguientes escalas: escala de Hamilton para la Depresión de 21 ítems, Montgomery-Asberg para la depresión, Hamilton para la Ansiedad, Impresión Clínica Global, Inventario de Depresión de Beck, escala Columbia para el riesgo de suicidio, Yale-Brown para síntomas obsesivo-compulsivos, valoración cognitiva (exclusivamente basal y a los 6 meses) y listado de efectos adversos.

3. Describir la utilidad del uso conjunto de marcadores farmacogenéticos relacionados con la respuesta antidepresiva (principalmente isoformas CYP2B6 y CYP2C19) y la monitorización farmacocinética en la optimización farmacológica del tratamiento del TDM de esta muestra de pacientes.

4. Describir la influencia de antecedentes familiares, hábitos tóxicos, estilos de vida, condicionantes sociales, laborales y familiares en la evolución del TDM de esta muestra de pacientes.

5. Determinar la implicación de las medidas no farmacológicas en la evolución del TDM de esta muestra de pacientes (medidas higiénico-dietéticas de estilo de vida, psicoterapia, terapia electroconvulsiva).

**9. MÉTODOS DE INVESTIGACIÓN**

**a) Diseño del estudio**:

Estudio observacional, prospectivo a 6 meses de pacientes con TDM que por práctica clínica precisan determinaciones séricas de sertralina, analizando variables que son recogidas por práctica clínica habitual. Se ha optado por este diseño por no suponer ningún riesgo médico añadido para los participantes y para estudiar la hipótesis del estudio en condiciones de práctica clínica real. Se realizará un protocolo prospectivo de recogida de datos a partir del inicio del estudio de variables que se recogen en la historia psiquiátrica por práctica habitual (lista completa en apartado de variables). Se recogerán variables generales (fecha de ingreso, etc.) antropométricas (edad, peso, talla, superficie corporal, etc.), clínicas (diagnóstico principal, antecedentes médicos, etc.), analíticas (pruebas hepáticas, urea y creatinina plasmáticas, ionograma, etc.), de tratamiento (dosis, hora de administración, comedicación, etc.), de eficacia (resultados de las escalas de intensidad sintomática Hamilton Depression (HAMD), Hamilton Anxiety (HAMA), Clinical global impression (CGI) scale, y restantes incluidas en el apartado de recogida de datos) y de seguridad (reacciones adversas y toxicidad).

Además, se realizará un estudio observacional retrospectivo de las determinaciones de sertralina realizadas hasta el momento, incluyendo las variables del protocolo que se dispongan con la mayor aproximación temporal posible a los momentos establecidos en el protocolo. Se asume que la parte retrospectiva no se ajustará totalmente al protocolo en cuanto a que es posible que algunas variables no hayan sido recogidas o se hayan recogido en momentos distintos a los que marca el protocolo. A pesar de ello, se considera de utilidad la recogida de estos datos retrospectivos especialmente para las variables primarias. Se han incluido unas ventanas temporales para tratar de homogeneizar la recogida retrospectiva.

Para garantizar que la realización del estudio prospectivo no modifique los hábitos de prescripción médica se ha establecido como criterio de inclusión un diagnóstico principal para el cual la sertralina supone un tratamiento farmacológico de elección y un criterio temporal (6 meses) inferior al periodo mínimo habitual de duración del tratamiento.

Los datos se obtendrán mediante la revisión de historias clínicas en el sistema de información de la CUN. Los investigadores del estudio garantizarán la confidencialidad de los datos de los sujetos y velarán porque se cumpla en todo momento con lo establecido en la ley 15/1999, de protección de datos de carácter personal y el RD 1720/2007.

**b) Entorno**:

Se ha optado por criterios de inclusión y exclusión amplios que permitan la inclusión de pacientes representativos de la práctica clínica real. Además de la rama prospectiva, se incluye una rama retrospectiva para conseguir un mayor tamaño muestral, para maximizar la representatividad y la potencia estadística.

La población del estudio consiste en pacientes con con sintomatología depresiva primaria o secundaria en tratamiento farmacológico antidepresivo con sertralina del departamento de Psiquiatría de la Clínica Universidad de Navarra, con los siguientes criterios de inclusión y sin el criterio de exclusión:

Criterios de inclusión/exclusión

Inclusión

Pacientes de cualquier edad en tratamiento con sertralina por presentar sintomatología depresiva primaria o secundaria a otros diagnósticos psiquiátricos o médicos.

Exclusión

Sospecha de falta de adherencia al tratamiento. Triple confirmación de adherencia: prescripción médica, referida verbalmente por el paciente, referida verbalmente por acompañante del paciente (cuando sea posible y con previa autorización del paciente).

Los pacientes se seleccionarán a partir de las prescripciones consecutivas de sertralina del sistema informatizado e historias clínicas, en las ramas prospectiva (como mínimo hasta que se alcance el tamaño muestral requerido) y retrospectiva (sin una fecha límite retrospectiva) que reúnan los criterios de inclusión y no reúnan el criterio de exclusión.

**c) Variables**:

**De exposición:**

-Variables farmacocinéticas (basal, a los 15 días, 30 días, 60 días, 90 días y 6 meses): Fecha de extracción, hora de extracción, concentraciones séricas de sertralina y n-desmetilsertralina.

**De resultados primarios:**

-Variables de eficacia (basal, a los 15 (+/-2) días, 30 (+/-5) días, 60(+/-10) días, 90(+/-15) días y 180 (+/-15) días): a través de la escala de Hamilton para la Depresión de 21 ítems.

**De resultados secundarios:**

-Diagnóstico principal (basal): tipo de trastorno depresivo mayor unipolar.

-Comorbilidades psiquiátricas y médicas generales (basal)

-Variables antropométricas, demográficas y hábitos de vida (basal, antes del inicio de tratamiento): Sexo, edad, estado civil, lugar en la fratria, talla, peso, IMC, superficie corporal, práctica de ejercicio físico (intensidad, frecuencia), dieta (saludable/no saludable), grado de formación escolar, profesional o académica, actividad de cuidado familiar (cuidado de hijos o familiares dependientes), actividad laboral (sí/no), creencias/prácticas religiosas (sí/no), relaciones sociales (mantenida/empobrecida/nula-soledad), residencia (urbana/rural).

-Variables de tratamiento farmacológico (basal, a los 15 (+/-2) días, 30 (+/-5) días, 60(+/-10) días, 90(+/-15) días y 180 (+/-15) días) : Dosis, intervalo de dosificación, hora de administración, duración de tratamiento con esa dosis, comedicación incluyendo su posología y duración. En la rama prospectiva se realizará una triple confirmación de cumplimiento de adherencia: prescripción médica, referida verbalmente por el paciente, referida verbalmente por acompañante del paciente (cuando sea posible y con previa autorización del paciente).

-Variables de eficacia (basal, a los 15 (+/-2) días, 30 (+/-5) días, 60(+/-10) días, 90(+/-15) días y 180 (+/-15) días) : a través de las siguientes escalas de intensidad sintomática: Montgomery-Asberg para la depresión, Hamilton para la Ansiedad, Impresión Clínica Global, Inventario de Depresión de Beck, escala Columbia para el riesgo de suicidio, Yale-Brown para síntomas obsesivo-compulsivos, valoración cognitiva (exclusivamente basal y a los 6 meses).

-Variables de tolerabilidad (basal, a los 15 (+/-2) días, 30 (+/-5) días, 60(+/-10) días, 90(+/-15) días y 180 (+/-15) días) : recogida de efectos adversos: listado y graduación.

-Variables de tratamiento no farmacológico (basal, a los 15 (+/-2) días, 30 (+/-5) días, 60(+/-10) días, 90(+/-15) días y 180 (+/-15) días) : Medidas higiénico-dietéticas de estilo de vida (higiene del sueño, patrón de dieta saludable, ejercicio físico regular, abandono de tabaco y/o tóxicos). Tipo de psicoterapia, Cantidad/frecuencia de sesiones de psicoterapia, terapia electroconvulsiva (tipo y frecuencia).

-Variables analíticas (al inicio de tratamiento y posteriormente hasta los 180 (+/-15 días, si existen): Hemograma, ionograma, Vit. B1, B6, B12, folato, ferritina, albumina plasmática, ALT, AST, bilirrubina total y directa, fosfatasa alcalina, urea y creatinina.

-Variables genéticas (si se cuenta con análisis farmacogenético por criterio clínico): isoformas CYP, principalmente isoformas CYP2B6 y CYP2C19

-Conducta o ideación suicida (a lo largo de la vida, basal, a los 15 (+/-2) días, 30 (+/-5) días, 60(+/-10) días, 90(+/-15) días y 180 (+/-15) días)

-Hábitos tóxicos (a lo largo de la vida, basal, a los 15 (+/-2) días, 30 (+/-5) días, 60(+/-10) días, 90(+/-15) días y 180 (+/-15) días): consumo de alcohol, tabaco u otros tóxicos, adicciones conductuales

-Otras variables (basal, a los 15 (+/-2) días, 30 (+/-5) días, 60(+/-10) días, 90(+/-15) días y 180 (+/-15) días) : antecedentes psiquiátricos familiares, acontecimientos vitales estresantes-precipitantes/traumáticos (también a lo largo de la vida), situación familiar de dependencia (también a lo largo de la vida), expectativas sobre el tratamiento farmacológico, conciencia de enfermedad.

d) Fuentes de datos:

Todas las variables analizadas se recogen en la historia psiquiátrica por práctica habitual. Los datos se obtendrán mediante la revisión de historias clínicas en el sistema electrónico de historias clínicas de la CUN. Las muestras para la determinación de concentraciones séricas de sertralina y n-desmetilsertralina proceden de extracción nueva de pacientes y su destino final después de finalizado el estudio es la destrucción.

**e) Tamaño muestral**

Se ha estimado un tamaño de muestra necesario para realizar el estudio de al menos 61 pacientes para alcanzar una potencia estadística del 80% para detectar diferencias considerando un coeficiente de correlación de 0,35 frente a una hipótesis nula de no correlación, considerando un test de hipótesis a dos colas y un nivel de significación del 5%.

**f) Gestión de los datos**.

Sólo tendrán acceso a los datos de la historia clínica necesarios para alcanzar los objetivos del proyecto las personas designadas por el promotor, el monitor, el investigador y su equipo de colaboradores y las autoridades sanitarias pertinentes.

El promotor y los investigadores del estudio deben garantizar la confidencialidad de los datos de los sujetos y velar porque se cumpla en todo momento con lo establecido en la ley 3/2018, de protección de datos de carácter personal y el Reglamento (UE) 2016/679 del Parlamento europeo y del Consejo de 27 de abril de 2016 de Protección de Datos.

**g) Análisis de los datos.**

Se realizará un análisis descriptivo incluyendo el cálculo de la media y la desviación estándar para variables cuantitativas, y de porcentajes para variables cualitativas. Se empleará el coeficiente de correlación de Pearson para valorar la asociación entre la concentración plasmática de sertralina y el grado de intensidad de los síntomas depresivos. Se calculará la odds ratio y su respectivo intervalo de confianza al 95% como medida de asociación utilizando modelos de regresión logística. Los valores p<0.05 serán considerados para establecer la significación estadística. Los análisis estadísticos serán realizados con el programa Stata 14 (StataCorp. 2015. Stata Statistical Software: Release 14. College Station, TX: StataCorp LP).

**h) Control de calidad.**

El control de los datos recogidos en el CRD estará asegurado por propio equipo investigador. Los investigadores principales controlarán el seguimiento del protocolo del estudio.

**i) Limitaciones de los métodos de investigación.**

Este estudio tiene las limitaciones propias de una metodología observacional, con menor capacidad para establecer inferencias causales que un ensayo clínico. Para controlar esta limitación, se ha diseñado la recogida de numerosas variables para recoger suficiente información para poder ajustar por los factores de confusión más relevantes. Además, en la rama retrospectiva es probable que los datos disponibles no se ajusten totalmente al protocolo y haya que recurrir a los datos recogidos en las mejores aproximaciones temporales posibles a los momentos establecidos en el protocolo prospectivo. Para controlar esta limitación, de inicio se realizará un análisis independiente de los datos retrospectivos y sólo si la calidad de estos datos retrospectivos es suficiente, pasarán a analizarse conjuntamente con los datos prospectivos.

**10. PROTECCIÓN DE LAS PERSONAS SOMETIDAS AL ESTUDIO**

**a) Evaluación beneficio-riesgo para los sujetos de investigación, en su caso.**

No procede, ya que en este estudio se analizan variables recogidas en la historia clínica por práctica clínica habitual.

**b) Consideraciones sobre información a los sujetos y consentimiento informado.**

Una vez explicado el estudio en su totalidad al paciente, se obtendrá su consentimiento informado por escrito o bien de su tutor o representante legal antes de hacer efectiva su participación en el estudio.

El investigador (o persona delegada) firmará y fechará también el formulario de consentimiento. El investigador archivará el Formulario de Consentimiento original firmado en el Archivo del Investigador del centro.

El paciente recibirá el Formulario de Consentimiento Informado y se le informará de que la participación en el estudio es voluntaria y puede retirarse en cualquier momento sin perjuicio de su asistencia médica posterior. Ni la Hoja de Información del Paciente ni el Consentimiento Informado pueden modificarse sin el acuerdo del Comité de Ética de la investigación con medicamentos y del promotor.

Un ejemplar del Formulario de Consentimiento Informado debe estar en poder del paciente.

El Formulario de Consentimiento incluye la información relativa a la necesidad de acceder las Historias Clínicas.

**c) Confidencialidad de los datos:**

Con el fin de garantizar la confidencialidad de los datos; se identificará a los pacientes del estudio con un número, de forma correlativa según el orden de inclusión. Los cuadernos de recogida de datos (CRD), informes y comunicaciones del estudio irán identificados con dicho número. Sólo tendrán acceso al material identificado necesario para alcanzar los objetivos del estudio el promotor, el investigador y su equipo de colaboradores, el Comité de Ética de la Investigación con medicamentos que tutela el estudio y las autoridades sanitarias pertinentes, según autorización del paciente.

El contenido de los CRD así como los documentos generados durante el estudio serán custodiados de usos no permitidos por personas ajenas al estudio, y por tanto, serán considerados estrictamente confidenciales y no serán revelados a terceros excepto los especificados en el párrafo anterior.

El promotor y los investigadores del estudio deben garantizar la confidencialidad de los datos de los sujetos y velar porque se cumpla en todo momento con lo establecido en la ley 3/2018, de protección de datos de carácter personal y el reglamento europeo (UE) 2016/679.

**d) Interferencia con los hábitos de prescripción del médico**

Para garantizar que la realización del estudio prospectivo no modifique los hábitos de prescripción médica se ha establecido como criterio de inclusión un diagnóstico principal para el cual la sertralina supone un tratamiento farmacológico de elección y un criterio temporal (6 meses) inferior al periodo mínimo habitual de duración del tratamiento.

**11. GESTIÓN Y NOTIFICACIÓN DE REACCIONES ADVERSAS y demás eventos relevantes que** **aparezcan durante el estudio**

Los casos de sospechas de reacciones adversas graves e inesperadas a sertralina se comunicarán por el cauce oficial previsto en la CUN. El equipo investigador recogerá información de tolerancia de interés para el estudio.

DEFINICIONES

Acontecimiento adverso se define como cualquier incidencia perjudicial para la salud en un paciente o sujeto de un estudio clínico tratado con un medicamento, aunque no tenga necesariamente una relación causal con dicho tratamiento.

Un AA puede ser, por tanto, cualquier signo desfavorable y no intencionado (incluyendo un hallazgo anormal de laboratorio), síntoma o enfermedad temporalmente asociada con el uso de un medicamento en investigación, esté o no relacionado con el medicamento en investigación.

Acontecimiento adverso grave se define como cualquier acontecimiento adverso o reacción adversa que, a cualquier dosis:

- Provoque la muerte del paciente.
- Amenace la vida del paciente (en opinión del investigador, el paciente en el momento del AA o RA está en un riesgo real de muerte; no se refiere a que el AA/RA hipotéticamente pudiera haber ocasionado la muerte en el caso de haber sido más intenso).
- Requiera la hospitalización o prolongación de la hospitalización del paciente
- Provoque invalidez o incapacidad permanente o importante
- Dé lugar a una anomalía o malformación congénita

Reacción adversa se considera a cualquier reacción nociva y no intencionada a un medicamento en investigación, independientemente de la dosis administrada. A diferencia de un AA, en el caso de una reacción adversa existe una sospecha de relación causal entre el medicamento en investigación y el acontecimiento adverso.

La determinación de la posible relación con el tratamiento del estudio deberá realizarse de acuerdo con las siguientes definiciones:

- NO RELACIONADO: No hay evidencia de ninguna relación causal.
- IMPROBABLE: Existe poca evidencia que sugiera una relación causal (p.ej. el acontecimiento no se presentó en un período de tiempo razonable después de la administración del medicamento/procedimiento del estudio). Hay otra explicación razonable para el acontecimiento (p.ej. la condición clínica del paciente, otros tratamientos concomitantes).
- POSIBLE: Existen evidencias que sugieren una posible relación causal (p.ej. porque el acontecimiento ocurrió en un tiempo razonable después de la administración del medicamento del estudio). Sin embargo, la influencia de otros factores puede haber contribuido al acontecimiento (p.ej. la condición clínica del paciente, otros tratamientos concomitantes).
- PROBABLE: Existen evidencias que sugieren una relación causal y la influencia de otros factores es poco probable.
- DEFINITIVO: Existen evidencias claras que sugieren una relación causal y se puede descartar una posible contribución de otros factores.

Reacción Adversa Grave e Inesperada (RAGI). Cualquier reacción adversa grave cuya naturaleza, intensidad o consecuencias no se corresponde con la información de referencia para el medicamento.

El carácter inesperado de una reacción adversa se basa en el hecho de no haber sido observado previamente y no se basará en lo que pudiera ser anticipado en función de las propiedades farmacológicas del medicamento.

COMUNICACIÓN DE ACONTECIMIENTOS ADVERSOS.

Los casos de reacciones adversas graves e inesperadas a sertralina deben se registrados sistemáticamente por el profesional sanitario y transmitidos a las autoridades por el cauce oficial estblecido para ello en la CUN. Este estudio no modifica la pauta terapéutica de los participantes, lo cual incluye las obligaciones inherentes a la administración de medicamentos y la detección y comunicación de los mismos a los sistemas de farmacovigilancia cuando proceda.

El profesional responsable del tratamiento será el encargado de comunicar las sospechas de reacciones adversas cuando proceda a través del cauce habituales establecido para ello en la CUN.

**12. PLAN DE TRABAJO**

(tareas, hitos y cronología del estudio).

| **Actividad** | **Fecha inicio** | **Fecha fin** |
| --- | --- | --- |
| Recogida de datos | Julio 2021 | Diciembre 2026 |
| Análisis e interpretación de los datos obtenidos | enero 2022 | Febrero 2027 |
| Redacción | Marzo 2022 | Junio 2027 |
| Informe final de resultados | Septiembre 2027 |  |

**13. PLANES DE DIFUSIÓN Y COMUNICACIÓN DE LOS RESULTADOS DEL ESTUDIO.**

Los resultados se enviarán para su publicación a revistas científicas especializadas con revisión por pares.

**14. REFERENCIAS.**

1. Mauri MC, Laini V, Cerveri G, Scalvini ME, Volonteri LS, Regispani F, et al. Clinical outcome and tolerability of sertraline in major depression: A study with plasma levels. Prog Neuro-Psychopharmacology Biol Psychiatry [Internet]. 2002 [cited 2021 Apr 26];26(3):597–601. Available from: https://pubmed.ncbi.nlm.nih.gov/11999914/

2. Mauri MC, Fiorentini A, Cerveri G, Volonteri LS, Regispani F, Malvini L, et al. Long-term efficacy and therapeutic drug monitoring of sertraline in major depression. Hum Psychopharmacol [Internet]. 2003 Jul [cited 2021 Apr 24];18(5):385–8. Available from: https://pubmed.ncbi.nlm.nih.gov/12858326/

3. Merikangas K, Rihmer Z. 13.2 Mood Disorders: Epidemiology. In: Kaplan and Sadock’s Comprehensive Textbook of Psychiatry, Tenth Edition. 2017. p. 1614–9.

4. Ayuso-Mateos JL, Vázques-Barquero JL, Dowrick C, Lehtinen V, Dalgard OS, Casey P, et al. Depressive disorders in Europe: Prevalence figures from the ODIN study. Br J Psychiatry [Internet]. 2001 [cited 2021 May 7];179(OCT.):308–16. Available from: https://pubmed.ncbi.nlm.nih.gov/11581110/

5. Akiskal HS. 13.4 Mood Disorders: Clinical Features. In: Sadock B, Sadock V, Ruiz P, editors. Kaplan and Sadock’s Comprehensive Textbook of Psychiatry, Tenth Edition. 2017. p. 1630–60.

6. Sánchez-Villegas A, Lahortiga F, Molero P, Martínez González MA (Ed. ). Capítulo 22 Depresión y otras enfermedades mentales. En: Conceptos de Salud Pública y Estrategias Preventivas Un Manual para Ciencias de la Salud. In Elsevier; 2018 [cited 2018 Dec 8]. Available from: https://tienda.elsevier.es/conceptos-de-salud-publica-y-estrategias-preventivas-9788491131205.html?gclid=EAIaIQobChMIs6uxluuQ3wIVk_hRCh2iRQU-EAQYASABEgIhgfD_BwE

7. National Institute for Health and Care Excellence. Depression in adults: recognition and management | Guidance | NICE [Internet]. [cited 2021 May 7]. Available from: https://www.nice.org.uk/guidance/CG90

8. Rush AJ, Trivedi MH, Wisniewski SR, Nierenberg AA, Stewart JW, Warden D, et al. Acute and longer-term outcomes in depressed outpatients requiring one or several treatment steps: a STAR*D report. Am J Psychiatry [Internet]. 2006 Nov [cited 2017 Nov 5];163(11):1905–17. Available from: http://psychiatryonline.org/doi/abs/10.1176/ajp.2006.163.11.1905

9. Yatham LN, Kennedy SH. 13.7 Mood Disorders: Pharmacological Treatment of Depression and Bipolar Disorders. In: Kaplan and Sadock’s Comprehensive Textbook of Psychiatry, Tenth Edition. 2017. p. 1676–700.

10. Pompili M, Venturini P, Palermo M, Stefani H, Seretti ME, Lamis DA, et al. Mood disorders medications: Predictors of nonadherence - Review of the current literature [Internet]. Vol. 13, Expert Review of Neurotherapeutics. Expert Rev Neurother; 2013 [cited 2021 May 14]. p. 809–25. Available from: https://pubmed.ncbi.nlm.nih.gov/23898852/

11. Melfi CA, Chawla AJ, Croghan TW, Hanna MP, Kennedy S, Sredl K. The effects of adherence to antidepressant treatment guidelines on relapse and recurrence of depression. Arch Gen Psychiatry [Internet]. 1998 [cited 2021 May 14];55(12):1128–32. Available from: https://pubmed.ncbi.nlm.nih.gov/9862557/

12. Rosenblat JD, Lee Y, McIntyre RS. The effect of pharmacogenomic testing on response and remission rates in the acute treatment of major depressive disorder: A meta-analysis. Vol. 241, Journal of Affective Disorders. Elsevier B.V.; 2018. p. 484–91.

13. Zeier Z, Carpenter LL, Kalin NH, Rodriguez CI, McDonald WM, Widge AS, et al. Clinical implementation of pharmacogenetic decision support tools for antidepressant drug prescribing. Am J Psychiatry [Internet]. 2018 Sep 1 [cited 2021 May 17];175(9):873–86. Available from: www.pharmgkb.org

14. Hiemke C, Bergemann N, Clement HW, Conca A, Deckert J, Domschke K, et al. Consensus Guidelines for Therapeutic Drug Monitoring in Neuropsychopharmacology: Update 2017 [Internet]. Vol. 51, Pharmacopsychiatry. Georg Thieme Verlag; 2018 [cited 2021 May 7]. p. 9–62. Available from: https://pubmed.ncbi.nlm.nih.gov/28910830/
