# Supplementary material for: The PREDEP-SERT study protocol: A 6-month follow-up cohort study of predictors of effectiveness, tolerability and safety of sertraline for depression using Therapeutic Drug Monitoring
Source: PLoS One. 2025 Aug 8;20(8):e0325335. doi: 10.1371/journal.pone.0325335 (PMC12333983; doi:10.1371/journal.pone.0325335)
Supplement: S2 File — (DOCX) [file pone.0325335.s002.docx]

**PROTOCOL**

**1. DESCRIPTIVE TITLE AND PROTOCOL VERSION**

Study of Predictors of Efficacy and Tolerability of Antidepressant Treatment in Patients with Major Depression Treated with Sertraline.

Version #3, March 6th, 2024

**2. STUDY SPONSOR**

CLINICA UNIVERSIDAD DE NAVARRA

Avenida Pío XII, 36

31008 Pamplona, Spain

**3. STUDY TEAM.**

Azucena Aldaz Pastor. PhD in Pharmacy. Specialist in Hospital Pharmacy. Clínica Universidad de Navarra.

Felipe Ortuño Sánchez Pedreño, PhD in Medicine, Specialist in Psychiatry. Clínica Universidad de Navarra.

Begoña Tapia Alzuguren. PhD in Pharmacy. Specialist in Hospital Pharmacy. Clínica Universidad de Navarra.

Covadonga Canga-Espina, MD. Specialist in Psychiatry . Clínica Universidad de Navarra.

María del Mar Unceta González MD. Specialist Psychiatry specialist. Clínica Universidad de Navarra.

Enrique Aubá Guedea. PhD in Medicine. Specialist in Psychiatry. Clínica Universidad de Navarra.

José Pablo Bullard García Naranjo, MD. Specialist in Psychiatry. Clínica Universidad de Navarra.

Coordinator: Patricio Molero Santos, PhD in Medicine. Specialist in Psychiatry. Clínica Universidad de Navarra.

**4. SUMMARY**

a) **Title and Subtitles, with the version and date of the protocol, name and surname of the main author, and the organization for which he/she works**:

Title: Study of efficacy and tolerability Predictors of Efficacy and Tolerability of Antidepressant Treatment in Patients with Major Depression Diagnosis Treated with Sertraline.

Short title: Predictors of Response in Depression-SERT (PREDEP-SERT).

Version #3, March 6th, 2024

Co-Pis: Azucena Aldaz and Patricio Molero. Clínica Universidad de Navarra

**b) Justification and context**:

Major depressive disorder (MDD) is a severe, recurrent, and disabling illness with a high prevalence in our area. It is associated with increased morbidity and mortality due to the risk of suicide and other associated medical and psychiatric conditions. It is also one of the most important causes of disability adjusted life years (DALY) lost. Antidepressant treatment is a fundamental element of MDD treatment, and selective serotonin reuptake inhibitors (SSRIs) are recommended as the first line of treatment. During the first weeks of treatment, a dose adjustment is made to identify the patient's minimum effective dose, which combines efficacy and acceptable tolerability. SSRI dose adjustment is made based on the established dose ranges in the summary of product characteristics and clinical judgment. Certain genetic and environmental factors can facilitate the development of toxicity or lack of efficacy within the established dose ranges, requiring the use of lower or higher doses. These situations imply the risk of, respectively, toxicity or excessively prolonged response latency, which can be associated with clinical complications. These situations are usually managed based on individual clinical judgment. There is no established consensus nor clinical guidelines for personalized dose adjustments. Therefore, determination of plasma levels may be determinant in this regard. An example of an SSRI for which there is preliminary evidence of the importance of dose adjustment based on plasma levels is sertraline, with optimal ranges of low or intermediate plasma concentrations associated with greater efficacy. Hence, increases beyond certain doses, even if within the established dose ranges in the summary of product characteristics, would not provide any clinical benefit. Further evidence is needed regarding the optimal therapeutic range of this drug in terms of efficacy and tolerability. This study aims to investigate whether there are optimal sertraline plasma concentration ranges associated with therapeutic response and acceptable tolerability from the second week and in the first 6 months of treatment, and what clinically meaningful symptomatic, genetic, and environmental factors may predict this association.

**c) Research hypothesis and objectives**:

Working hypothesis:

There is an association between plasma sertraline concentration and the intensity of depressive symptoms after 6 months of treatment.

Primary objective:

To analyze the correlation between plasma sertraline concentration and depressive symptom severity, as measured by the 21-item Hamilton Depression Rating Scale (HDRS) after 6 months of treatment.

Secondary objectives:

To assess the overall clinical response, to both pharmacological and non-pharmacological treatment, and to identify possible genetic and environmental factors of treatment response in patients diagnosed with MDD according to the following variables:

1. To study the association between plasma sertraline concentration and response (which is a 50% decrease in the intensity of depressive symptoms reflected by the 21-item Hamilton Depression Rating Scale – HDRS –) of depressive symptoms after 6 months of treatment.
2. To study the association between plasma sertraline concentration and remission (score on the 21-item Hamilton Depression Rating Scale – HDRS – less or equal to 7) of depressive symptoms after 6 months of treatment.
3. To study the correlation between the improvement of global symptomatic profile and the tolerability and plasma concentrations of sertraline at 15 days, 30 days, 60 days, 90 days and 6 months of antidepressant treatment in patients with MDD assessed through the following scales: Hamilton Depression Scale of 21 items (HDRS), Montgomery-Asberg for depression (MADRS), Hamilton for Anxiety (HAM-A), Clinical Global Impression (CGI), Beck Depression Inventory (BDI), Columbia-Suicide Severity Rating Scale (C-SSRS), Yale-Brown Obsessive-Compulsive Scale (Y-BOCS), cognitive assessment (exclusively at baseline and after 6 months of treatment) and adverse effects.
4. To describe the usefulness of combining pharmacogenetic markers related to antidepressant response (mainly CYP2B6 and CYP2C19 isoforms) and pharmacokinetic monitoring in pharmacological optimization of MDD treatment in this sample of patients.
5. To determine the involvement of non-pharmacological measures in the evolution of MDD in this sample of patients, such as hygienic-dietary lifestyle measures, psychotherapy or electroconvulsive therapy.
6. To describe the influence of family history, drug use, lifestyle, social, work, and family factors on the evolution of MDD in this sample of patients.

**d) Study design:**

Observational, both prospective and retrospective.

**e) Population:**

Patients with primary or secondary depressive symptoms undergoing antidepressant pharmacological treatment with sertraline at the Department of Psychiatry in Clínica Universidad de Navarra, who meet the following inclusion criteria and do not meet any exclusion criteria:

Inclusion/Exclusion criteria

*Inclusion*

Patients of any age receiving treatment with sertraline for primary or secondary depressive symptoms due to other psychiatric or medical diagnoses.

*Exclusion*

Suspected non-adherence to treatment.

**f) Variables:**

- ***Anthropometric, demographic and lifestyle variables*** (baseline, before the start of treatment): Sex, age, marital/civil status, place in the phratry, height, weight, BMI, body surface area, physical exercise (intensity, frequency), diet (healthy/unhealthy), professional or academic education, family care responsabilities (care of children or dependent relatives), work activity (yes/no), religious beliefs/ community religious activity (yes/no), social relationships (maintained/impoverished/none-loneliness), residence (urban/rural).

- ***Main diagnosis*** (baseline).

- Psychiatric and medical ***comorbidities*** (baseline)

- ***Pharmacological treatment variables*** (baseline, after 15 (+/-2) days, 30 (+/-5) days, 60 (+/-10) days, 90 (+/-15) days and 180 (+/-15) days of treatment): Dose, dosing interval, time of administration, duration of treatment with that dose, co-medication (including its posology and duration). In the prospective arm, a triple confirmation of adherence will be performed: medical prescription, verbally reported by the patient, verbally reported by a patient's companion (when possible and with prior authorization from the patient).

-***Pharmacokinetic variables*** (baseline, after 15 (+/-2) days, 30 (+/-5) days, 60 (+/-10) days, 90 (+/-15) days, and 180 (+/-15) days of treatment): Date and time of extraction, serum concentrations of sertraline and n-desmethylsertraline.

- ***Efficacy variables*** (baseline, after 15 (+/-2) days, 30 (+/-5) days, 60 (+/-10) days, 90 (+/-15) days and 180 (+/-15) days of treatment): through the following symptom intensity scales: Hamilton for Depression (HAM-D), Montgomery-Asberg for Depression (MADRS), Hamilton for Anxiety (HAM-A), Clinical Global Impression (CGI), Beck Depression Inventory (BDI), Columbia scale for suicide risk (C-SSRS), Yale-Brown for obsessive-compulsive symptoms (Y-BOCS), cognitive assessment (exclusively baseline and at 6 months).

- ***Tolerability variables*** (baseline, after 15 (+/-2) days, 30 (+/-5) days, 60 (+/-10) days, 90 (+/-15) days, and 180 (+/-15) days of treatment): adverse effects (list and measurement).

- ***Non-pharmacological treatment variables*** (baseline, after 15 (+/-2) days, 30 (+/-5) days, 60 (+/-10) days, 90 (+/-15) days and 180 (+/-15) days of treatment): Hygienic-dietary lifestyle measures (sleep hygiene, healthy diet pattern, regular physical exercise, smoking and/or drug cessation). Type of psychotherapy, Number/frequency of psychotherapy sessions, electroconvulsive therapy (type and frequency).

-***Analytical variables*** (at baseline and subsequently up to 180 (+/-15) days, if they exist): Complete blood count, ionogram, vitamin B1, B6, B12, folate, ferritin, plasma albumin, ALT, AST, total and direct bilirubin, alkaline phosphatase, urea, and creatinine.

- ***Genetic variables*** (if pharmacogenetic analysis is available per clinical judgment): CYP isoforms, including CYP2B6 and CYP2C19 isoforms.

- ***Suicidal behavior and/or ideation*** (lifetime, baseline, after 15 (+/-2) days, 30 (+/-5) days, 60 (+/-10) days, 90 (+/-15) days, and 180 (+/-15) days of treatment).

- ***Toxic habits*** (throughout life, baseline, after 15 (+/-2) days, 30 (+/-5) days, 60 (+/-10) days, 90 (+/-15) days, and 180 (+/-15) days of treatment): consumption of alcohol, tobacco, or other toxins, as well as behavioral addictions.

-***Other variables*** (baseline, after 15 (+/-2) days, 30 (+/-5) days, 60 (+/-10) days, 90 (+/-15) days and 180 (+/-15) days of treatment): family psychiatric history, stressful-precipitating/traumatic life events (also throughout life), family dependency situation (also throughout life), expectations about pharmacological treatment, and awareness of illness.

**g) Data sources:**

All analyzed variables are collected in the psychiatric medical history as standard practice. Data will be obtained by reviewing medical records in Clínica Universidad de Navarra (CUN)'s electronic medical records system.

**h) Study sample size**:

A sample size of at least 61 patients has been estimated to achieve a statistical power of 80% to detect differences, considering a correlation coefficient of 0.35 against a null hypothesis of no correlation, bearing in mind a two-tailed hypothesis test and a significance level of 5%.

**i) Data analysis:**

A descriptive analysis will be performed, including the calculation of the mean and standard deviation for quantitative variables and percentages for qualitative variables. Pearson's correlation coefficient will be used to assess the association between plasma sertraline concentration and the intensity of depressive symptoms. The odds ratio and its 95% confidence interval will be calculated as a measure of association using logistic regression models. P values ​​<0.05 will be considered to establish statistical significance. Statistical analyses will be performed using Stata 14 (StataCorp. 2015. Stata Statistical Software: Release 14. College Station, TX: StataCorp LP).

**j) Stages and calendar:**

| **Activity** | **Start date** | **End date** |
| --- | --- | --- |
| Data collection | July 2021 | December 2026 |
| Analysis and interpretation of the data obtained | January 2022 | February 2027 |
| Drafting | March 2022 | June 2027 |
| Final results report | September 2027 |  |

**5. MODIFICATIONS AND UPDATES**

Any substantial modification to the study protocol after the start of data collection, must include justification, date, and indication of the affected section of the protocol.

**6. STAGES**

Summary with the planned schedule for at least the following stages:

a) Start of data collection.

b) End of data collection.

c) Study status reports, if applicable (Not applicable).

d) Interim reports on study results, if applicable (Not applicable).

e) Final report on study results.

| **Activity** | **Start date** | **End date** |
| --- | --- | --- |
| Data collection | July 2021 | December 2026 |
| Analysis and interpretation of the data obtained | January 2022 | February 2027 |
| Drafting | March 2022 | June 2027 |
| Final results report | September 2027 |  |

**7. JUSTIFICATION AND CONTEXT**

Major depressive disorder (MDD) according to DSM-5 (categories of moderate or severe depressive episodes according to ICD-10) is a serious, recurrent and disabling disease with a high prevalence in our environment, with estimates of the prevalence-half-life of 14.6% (3), and global prevalence in Europe of 8.56% (4)), which causes a deterioration in general medical health, social and family functioning, work performance, and quality of life. It is associated with an increase in morbidity and mortality due to the risk of suicide and other associated medical and psychiatric causes(5). It is one of the most important causes of disability-adjusted life years lost (DALYs) worldwide (the third-fourth in our environment), and it is estimated that it will be the first cause of DALYs worldwide in 2030(6).

MDD treatment consists of a combination of antidepressant pharmacotherapy and psychotherapy, and electroconvulsive therapy in life-threatening, severe, and/or refractory cases requiring a rapid response (7). Antidepressant therapy is a fundamental part of MDD management, and selective serotonin reuptake inhibitors (SSRI)(7), are recommended as first line of treatment. This class of medications is widely used to treat MDD. It is relatively safe but has the disadvantage of a prolonged response latency until the onset of significant clinical improvement (up to 5-8 weeks)(8). During the first weeks of treatment, a dose titration is performed to identify the patient's minimum effective dose (MED), which combines efficacy (antidepressant response: reduction of at least 50% of the intensity of pre-treatment symptoms on specific scales(9)) and acceptable tolerability. Doses lower than the MED are associated with a risk of MDD complications due to depressive worsening, while higher doses may be associated with a greater risk of adverse effects that may lead to a decrease in treatment adherence (10), a phenomenon that usually occurs in the first weeks and is associated with a greater risk of depressive relapse or recurrence (11). Currently, SSRI dose adjustments are based on product characteristics summary and clinical judgment. However, there is growing evidence that certain genetic factors may facilitate the development of toxicity or lack of efficacy at established dose ranges in some situations, necessitating the use of lower or higher doses (12,13). These situations are usually managed based on individual clinical judgment (exceptional, with pharmacogenetic guidance), and there is no established consensus in clinical guidelines that allows for personalized dose adjustments. Although determining plasma levels of antidepressants can be very useful in the management of some non-responders or toxic cases, optimal plasma concentration ranges are currently unknown. An example of an SSRI for which there is preliminary evidence of the importance of dose adjustment based on plasma levels is sertraline (1,2,14), with mixed results: on the one hand, a consensus has been established on a wide therapeutic range (10-150ng/ml) (14), although there is preliminary evidence of a curvilinear relationship between clinical improvement and plasma concentration, with greater efficacy in low or intermediate plasma concentration ranges (25-50ng/ml or 40-70ng/ml) (1,2), so that increases beyond certain doses, even if permitted by the the product technical datasheet, would not provide any clinical benefit. Clarifying this aspect is clinically important, since dose increases are linked to lengthening the already prolonged response latency of SSRIs, which can be associated with worsening or complications of MDD due to a loss of the opportunity to switch to a more effective and better-tolerated treatment (drug enhancement or switch). Furthermore, certain genetic polymorphisms of the enzyme groups involved in the first-pass hepatic metabolism of sertraline (CYP3A4, CYP2C19, and CYP2B6) can influence its plasma concentrations, and this enzymatic activity can be modified by certain drug interactions. Therefore, greater evidence is needed regarding the optimal therapeutic range for sertraline in terms of efficacy and tolerability, as well as the role of pharmacokinetics as a key modulator of the relationship between pharmacogenetic markers associated with this drug and its efficacy and tolerability.

This study aims to investigate whether there are optimal sertraline plasma concentration ranges associated with therapeutic response and acceptable tolerability from the second week and into the first 6 months of treatment, and what clinically useful symptomatic, pathophysiological, genetic, and environmental determinants can modify them.

**8. HYPOTHESIS AND RESEARCH OBJECTIVES**

**HYPOTHESIS**

There is an association between the plasma sertraline concentration and the degree of intensity of depressive symptoms after 6 months of treatment.

**PRIMARY AND SECONDARY OBJECTIVES.**

***Primary objective***: To study the correlation between sertraline plasma concentration and the intensity of depressive symptoms using the 21-item Hamilton Depression Rating Scale (HDRS) after 6 months of treatment.

***Secondary objectives***: To assess the overall clinical response to comprehensive treatment, both pharmacological and non-pharmacological, and to identify the genetic and environmental determinants of treatment response in patients diagnosed with MDD according to the following variables:

1. To study the association between sertraline plasma concentration and response (50% decrease in depressive symptoms intensity ousing the 21-item Hamilton Depression Rating Scale – HDRS –) and remission (score in the 21-item Hamilton Depression Rating Scale – HDRS – less than or equal to 7) of depressive symptoms after 6 months of treatment.

2. To study the correlation between the improvement in the global symptomatic profile and the tolerability and plasma concentrations of sertraline at 15 days, 30 days, 60 days, 90 days and 6 months in patients with MDD assessed through the following scales: Hamilton Depression Scale of 21 items (HDRS), Montgomery-Asberg for depression (MADRS), Hamilton for Anxiety (HAM-A), Clinical Global Impression (CGI), Beck Depression Inventory (BDI), Columbia Scale for suicide risk (C-SSRS), Yale-Brown for obsessive-compulsive symptoms (Y-BOCS) and cognitive assessment (exclusively baseline and at 6 months) and list of adverse effects.

3. To describe the utility of the combined use of pharmacogenetic markers related to antidepressant response (mainly CYP2B6 and CYP2C19 isoforms) and pharmacokinetic monitoring in the pharmacological optimization of MDD treatment in this sample of patients.

4. To describe the influence of family history, toxic habits, lifestyles, social, work, and family factors on the evolution of MDD in this sample of patients.

5. To determine the involvement of non-pharmacological measures in the evolution of MDD in this sample of patients (hygienic-dietary lifestyle measures, psychotherapy, electroconvulsive therapy).

**9. RESEARCH METHODS**

**a) Study design**:

A 6-month, prospective, observational study of patients with MDD who require sertraline serum levels as part of clinical practice. This design was chosen because it poses no additional medical risk to participants and allows the study hypothesis to be studied under real-life clinical practice conditions. A prospective data collection protocol will be implemented from the start of the study, using variables routinely collected from the psychiatric medical history (see the variables section for a full list). General variables (date of admission, etc.), anthropometric variables (age, weight, height, body surface area, etc.), clinical variables (main diagnosis, medical history, etc.), analytical variables (liver tests, plasma urea and creatinine, ionogram, etc.), treatment variables (dose, time of administration, co-medication, etc.), efficacy variables (results of the symptom intensity scales Hamilton Depression (HAMD), Hamilton Anxiety (HAM-A), Clinical global impression (CGI) scale, and the rest of the scales included in the data collection section), and safety variables (adverse reactions and toxicity) will be collected.

In addition, a retrospective observational study of sertraline measurements performed to date will be conducted, including the protocol variables available as close in time as possible to the protocol-established time points. It is assumed that the retrospective data will not fully adhere to the protocol, as some variables may not have been collected or may have been collected at different times than those specified in the protocol. Despite this, the collection of these retrospective data is considered useful, especially for the primary variables. Time windows have been included to ensure uniformity in the retrospective data collection.

To ensure that this prospective study does not alter medical prescribing habits, inclusion criteria have been established: a primary diagnosis for which sertraline is the pharmacological treatment of choice, and a time criterion (6 months) shorter than the usual minimum duration of treatment. Data will be obtained by reviewing medical records in the CUN (Clínica Universidad de Navarra) information system. The study researchers will guarantee the confidentiality of the subjects' data and will ensure compliance at all times with the provisions of Law 15/1999 on the protection of personal data and RD 1720/2007.

**b) Setting**:

Broad inclusion and exclusion criteria were chosen to ensure inclusion of patients representative of real-life clinical practice. In addition to the prospective arm, a retrospective arm was included to achieve a larger sample size and maximize representativeness and statistical power.

The study population consisted of patients with primary or secondary depressive symptoms receiving antidepressant pharmacological treatment with sertraline from the Department of Psychiatry at Clínica Universidad de Navarra, who met the following inclusion criteria and did not meet the exclusion criteria:

Inclusion/exclusion criteria

Inclusion

Patients of any age receiving sertraline treatment for primary or secondary depressive symptoms due to other psychiatric or medical diagnoses.

Exclusion

Suspected non-adherence to treatment. Triple confirmation of adherence: medical prescription, verbal referral by the patient, verbal referral by a patient's companion (when possible and with prior patient authorization).

Patients will be selected from consecutive sertraline prescriptions from the computerized system and medical records, in the prospective (at least until the required sample size is reached) and retrospective (without a retrospective cutoff date) arms, who meet the inclusion criteria and do not meet the exclusion criteria.

**c) Variables**:

**Exposure variables:**

- ***Pharmacokinetic variables*** (baseline, 15 days, 30 days, 60 days, 90 days, and after 6 months of treatment): Date of extraction, time of extraction, serum concentrations of sertraline and n-desmethylsertraline.

**Primary outcome variables:**

- ***Efficacy variables*** (baseline, after 15 (+/-2) days, 30 (+/-5) days, 60 (+/-10) days, 90 (+/-15) days, and 180 (+/-15) days): through the 21-item Hamilton Depression Scale (HDRS).

**Secondary outcome variables:**

-***Primary diagnosis*** (baseline): Unipolar major depressive disorder.

-Psychiatric and general medical ***comorbidities*** (baseline).

-***Anthropometric, demographic and lifestyle variables*** (baseline, before the start of treatment): Sex, age, marital status, presence of siblings, height, weight, BMI, body surface area, physical exercise (intensity, frequency), diet (healthy/unhealthy), level of education, professional or academic training, family care responsibilities (care of children or dependent relatives), work activity (yes/no), religious beliefs/practices (yes/no), social relationships (maintained/impoverished/none-loneliness), residence (urban/rural).

- ***Pharmacological treatment variables*** (baseline, after 15 (+/-2) days, 30 (+/-5) days, 60 (+/-10) days, 90 (+/-15) days and 180 (+/-15) days of treatment): Dose, dosing interval, time of administration, duration of treatment with that dose, co-medication (including its dosage and duration). In the prospective arm, Adherence will be confirmed through three sources: medical prescription, verbally reported by the patient, verbally reported by a patient's companion (when possible and with prior authorization from the patient).

- ***Efficacy variables*** (baseline, after 15 (+/-2) days, 30 (+/-5) days, 60 (+/-10) days, 90 (+/-15) days and 180 (+/-15) days of treatment): through the following symptom intensity scales: Hamilton for Depression (HAM-D), Montgomery-Asberg for Depression (MADRS), Hamilton for Anxiety (HAM-A), Clinical Global Impression (CGI), Beck Depression Inventory (BDI), Columbia scale for suicide risk (C-SSRS), Yale-Brown for obsessive-compulsive symptoms (Y-BOCS), cognitive assessment (exclusively baseline and at 6 months).

- ***Tolerability variables*** (baseline, after 15 (+/-2) days, 30 (+/-5) days, 60 (+/-10) days, 90 (+/-15) days, and 180 (+/-15) days of treatment): adverse effects (list and measurement).

- ***Non-pharmacological treatment variables*** (baseline, after 15 (+/-2) days, 30 (+/-5) days, 60 (+/-10) days, 90 (+/-15) days and 180 (+/-15) days of treatment): Hygienic-dietary lifestyle measures (sleep hygiene, healthy diet pattern, regular physical exercise, smoking and/or drug cessation). Type of psychotherapy, Number/frequency of psychotherapy sessions, electroconvulsive therapy (type and frequency).

-***Analytical variables*** (at baseline treatment and subsequently up to 180 (+/-15) days, if they exist): Complete blood count, ionogram, vitamin B1, B6, B12, folate, ferritin, plasma albumin, ALT, AST, total and direct bilirubin, alkaline phosphatase, urea, and creatinine.

- ***Genetic variables*** (if pharmacogenetic analysis is available based on clinical criteria): CYP isoforms, mainly CYP2B6 and CYP2C19 isoforms.

- ***Suicidal behavior or ideation*** (lifetime, baseline, after 15 (+/-2) days, 30 (+/-5) days, 60 (+/-10) days, 90 (+/-15) days, and 180 (+/-15) days of treatment).

- ***Toxic habits*** (throughout life, baseline, after 15 (+/-2) days, 30 (+/-5) days, 60 (+/-10) days, 90 (+/-15) days and 180 (+/-15) days of treatment): consumption of alcohol, tobacco or other toxics, also behavioral addictions.

-***Other variables*** (baseline, after 15 (+/-2) days, 30 (+/-5) days, 60 (+/-10) days, 90 (+/-15) days and 180 (+/-15) days of treatment): family psychiatric history, stressful-precipitating/traumatic life events (also throughout life), family dependency situation (also throughout life), expectations about pharmacological treatment, and awareness of illness.

**d) Data sources:**

All analyzed variables are collected as part of the standard practice in psychiatric medical history. Data will be obtained by reviewing medical records in Clínica Universidad de Navarra (CUN)'s electronic medical records system. The samples for the determination of serum concentrations of sertraline and n-desmethylsertraline come from new extractions from patients. After the study is completed results will be destroyed.

**e) Study sample size**:

A sample size of at least 61 patients has been estimated to achieve a statistical power of 80% for detecting differences, considering a correlation coefficient of 0.35 against a null hypothesis of no correlation. This assumes a two-tailed hypothesis test and sa ignificance level of 5%.

**f) Data Management**

Only those designated by the sponsor, the monitor, the researcher, their team of collaborators, and the relevant health authorities will have access to the medical record data necessary to achieve the project objectives.

The sponsor and study researchers will guarantee the confidentiality of the subjects' data and ensure compliance at all times with the provisions of Law 3/2018 on the protection of personal data and Regulation (EU) 2016/679 of the European Parliament and of the Council of April 27, 2016, on Data Protection.

**g) Data analysis:**

A descriptive analysis will be performed, including the calculation of the mean and standard deviation for quantitative variables and percentages for qualitative variables. Pearson's correlation coefficient will be used to assess the association between plasma sertraline concentration and the intensity of depressive symptoms. The odds ratio and its 95% confidence interval will be calculated as a measure of association using logistic regression models. P values ​​<0.05 will be considered to establish statistical significance. Statistical analyses will be performed using Stata 14 (StataCorp. 2015. Stata Statistical Software: Release 14. College Station, TX: StataCorp LP).

**h) Quality Control**

The data collected in the CRD will be monitored by the research team. The principal investigators will oversee compliance with the study protocol.

**i) Limitations of research methods:**

This study has the limitations inherent to an observational methodology, with a more limited capacity to establish causal inferences compared to a clinical trial. To address this limitation, the collection of numerous variables was designed to gather enough information to adjust for the most relevant confounding factors. Furthermore, in the retrospective arm, the available data may not fully adhere to the protocol, and it will be necessary to resort to data previously collected at the best possible temporal approximations to the time points established in the prospective protocol. To address this limitation, an independent analysis of the retrospective data will be performed initially, and only if the quality and homogeneity of these retrospective data are sufficient will they be analyzed jointly with the prospective data.

**10. PROTECTION OF INDIVIDUALS UNDERGOING STUDY**

**a) Benefit-risk assessment for research subjects, if applicable.**

Not applicable, as this study analyzes variables collected in the medical record as part of routine clinical practice.

**b) Considerations on information to subjects and informed consent**

Once the study has been fully explained to the patient, written informed consent will be obtained from them or their guardian or legal representative before participation in the study begins.

The researcher (or their delegate) will also sign and date the consent form. The researcher will file the original signed Consent Form in the center's Researcher's File.

The patient will receive the Informed Consent Form and will be informed that participation in the study is voluntary and that they may withdraw at any time without prejudice to their subsequent medical care. Neither the Patient Information Sheet nor the Informed Consent Form may be modified without the approval of the Drug Research Ethics Committee and the sponsor.

A copy of the Informed Consent Form must be kept by the patient.

The Consent Form includes information regarding the need to access medical records.

**c) Data confidentiality:**

To ensure data confidentiality, patients in the study will be identified by a sequential number, consecutively according to the order of inclusion. Data collection forms (DCFs), reports, and study communications will be identified with this number. Only the sponsor, the investigator, and their team of collaborators, the Drug Research Ethics Committee overseeing the study, and the relevant health authorities will have access to the identified material necessary to achieve the study objectives, with the patient's authorization.

The content of the DCFs, as well as the documents generated during the study, will be protected from unauthorized use by other individuals outside the study and, therefore, will be considered strictly confidential and will not be disclosed to third parties except as specified in the previous paragraph.

The sponsor and the study researchers must guarantee the confidentiality of the subjects' data and ensure compliance with the provisions of Law 3/2018 on the protection of personal data and European Regulation (EU) 2016/679.

**d) Interference with the physician's prescribing habits**

To ensure that the prospective study does not modify medical prescription habits, the inclusion criteria include a main diagnosis for which sertraline is a pharmacological treatment of first choice, and a follow-up period (6 months) shorter than the usual minimum duration of treatment has been established.

**11. MANAGEMENT AND REPORTING OF ADVERSE REACTIONS AND OTHER RELEVANT EVENTS**

Suspected cases of serious and unexpected adverse reactions to sertraline will be reported through the official channel provided to Clínica Universidad de Navarra (CUN) computerized system and medical records. The research team will collect tolerability-related information relevant to the study.

DEFINITIONS

An ***adverse event*** (AE) is defined as any adverse health event in a patient or clinical trial subject treated with a medicinal product, whether or not it is causally related to that treatment. An AE can therefore be any unintended adverse sign (including an abnormal laboratory finding), symptom, or illness temporarily associated with the use of an investigational medicinal product, whether or not related to the investigational medicinal product.

An ***adverse reaction*** (AR) is considered to be any harmful and unintended reaction to an investigational medicinal product, regardless of the dose administered. Unlike an AE, in the case of an AR, there is a suspected causal relationship between the investigational medicinal product and the adverse event.

The determination of a possible relationship with the study treatment should be made according to the following definitions:

- UNRELATED: No evidence of a causal relationship.

- IMPROBABLE: Little evidence suggests a causal relationship (e.g., the event did not occur within a reasonable time after administration of the study drug/procedure). There is another reasonable explanation for the event (e.g., the patient's clinical condition, other concomitant treatments).

- POSSIBLE: Evidence suggests a possible causal relationship (e.g., because the event occurred within a reasonable time after administration of the study drug). However, the influence of other factors may have contributed to the event (e.g., the patient's clinical condition, other concomitant treatments).

- PROBABLE: Evidence suggests a causal relationship, and the influence of other factors is unlikely.

- DEFINITIVE: There is clear evidence to suggest a causal relationship, and a possible contribution from other factors can be ruled out.

A ***serious adverse event*** (SAE) is defined as any adverse event or adverse reaction that, at any dose:

- Results in the death of the patient.

- Life-threatening (in the opinion of the investigator, the patient at the time of the AE or AR is at real risk of death; this does not mean that the AE or AR could hypothetically have resulted in death had it been more severe).

- Requires hospitalization or a prolongation of the patient's hospitalization.

- Causes permanent or significant disability or incapacity.

- Results in a congenital anomaly or malformation.

***Suspected Unexpected Serious Adverse Reaction*** (SUSAR). Any serious adverse reaction whose nature, intensity, or consequences do not align with the reference information for the medicinal product. The unexpected nature of an adverse reaction is based on the fact that it has not been previously observed and is not based on what might be anticipated based on the pharmacological properties of the medicinal product.

COMMUNICATION OF ADVERSE EVENTS

All cases of serious and unexpected adverse reactions to sertraline must be systematically recorded by healthcare professionals and reported to the authorities through the official channel established for this purpose by Clínica Universidad de Navarra (CUN). This study does not modify the therapeutic regimen of the participants, which includes the obligations inherent to the administration of medications and the detection and reporting of these to pharmacovigilance systems when appropriate.

The professional responsible for the treatment will be responsible for reporting suspected adverse reactions when appropriate through the usual channels established for this purpose by the CUN.

**12. WORK PLAN (tasks, milestones, and study timeline)**

| **Activity** | **Start date** | **End date** |
| --- | --- | --- |
| Data collection | July 2021 | December 2026 |
| Analysis and interpretation of the data obtained | January 2022 | February 2027 |
| Drafting | March 2022 | June 2027 |
| Final results report | September 2027 |  |

**13. PLANS FOR DIFFUSION AND COMMUNICATION OF THE STUDY RESULTS.**

The results will be submitted for publication to peer-reviewed scientific journals.

**14. REFERENCES.**

1. Mauri MC, Laini V, Cerveri G, Scalvini ME, Volonteri LS, Regispani F, et al. Clinical outcome and tolerability of sertraline in major depression: A study with plasma levels. Prog Neuro-Psychopharmacology Biol Psychiatry [Internet]. 2002 [cited 2021 Apr 26];26(3):597–601. Available from: https://pubmed.ncbi.nlm.nih.gov/11999914/

2. Mauri MC, Fiorentini A, Cerveri G, Volonteri LS, Regispani F, Malvini L, et al. Long-term efficacy and therapeutic drug monitoring of sertraline in major depression. Hum Psychopharmacol [Internet]. 2003 Jul [cited 2021 Apr 24];18(5):385–8. Available from: https://pubmed.ncbi.nlm.nih.gov/12858326/

3. Merikangas K, Rihmer Z. 13.2 Mood Disorders: Epidemiology. In: Kaplan and Sadock’s Comprehensive Textbook of Psychiatry, Tenth Edition. 2017. p. 1614–9.

4. Ayuso-Mateos JL, Vázques-Barquero JL, Dowrick C, Lehtinen V, Dalgard OS, Casey P, et al. Depressive disorders in Europe: Prevalence figures from the ODIN study. Br J Psychiatry [Internet]. 2001 [cited 2021 May 7];179(OCT.):308–16. Available from: https://pubmed.ncbi.nlm.nih.gov/11581110/

5. Akiskal HS. 13.4 Mood Disorders: Clinical Features. In: Sadock B, Sadock V, Ruiz P, editors. Kaplan and Sadock’s Comprehensive Textbook of Psychiatry, Tenth Edition. 2017. p. 1630–60.

6. Sánchez-Villegas A, Lahortiga F, Molero P, Martínez González MA (Ed. ). Capítulo 22 Depresión y otras enfermedades mentales. En: Conceptos de Salud Pública y Estrategias Preventivas Un Manual para Ciencias de la Salud. In Elsevier; 2018 [cited 2018 Dec 8]. Available from: https://tienda.elsevier.es/conceptos-de-salud-publica-y-estrategias-preventivas-9788491131205.html?gclid=EAIaIQobChMIs6uxluuQ3wIVk_hRCh2iRQU-EAQYASABEgIhgfD_BwE

7. National Institute for Health and Care Excellence. Depression in adults: recognition and management | Guidance | NICE [Internet]. [cited 2021 May 7]. Available from: https://www.nice.org.uk/guidance/CG90

8. Rush AJ, Trivedi MH, Wisniewski SR, Nierenberg AA, Stewart JW, Warden D, et al. Acute and longer-term outcomes in depressed outpatients requiring one or several treatment steps: a STAR*D report. Am J Psychiatry [Internet]. 2006 Nov [cited 2017 Nov 5];163(11):1905–17. Available from: http://psychiatryonline.org/doi/abs/10.1176/ajp.2006.163.11.1905

9. Yatham LN, Kennedy SH. 13.7 Mood Disorders: Pharmacological Treatment of Depression and Bipolar Disorders. In: Kaplan and Sadock’s Comprehensive Textbook of Psychiatry, Tenth Edition. 2017. p. 1676–700.

10. Pompili M, Venturini P, Palermo M, Stefani H, Seretti ME, Lamis DA, et al. Mood disorders medications: Predictors of nonadherence - Review of the current literature [Internet]. Vol. 13, Expert Review of Neurotherapeutics. Expert Rev Neurother; 2013 [cited 2021 May 14]. p. 809–25. Available from: https://pubmed.ncbi.nlm.nih.gov/23898852/

11. Melfi CA, Chawla AJ, Croghan TW, Hanna MP, Kennedy S, Sredl K. The effects of adherence to antidepressant treatment guidelines on relapse and recurrence of depression. Arch Gen Psychiatry [Internet]. 1998 [cited 2021 May 14];55(12):1128–32. Available from: https://pubmed.ncbi.nlm.nih.gov/9862557/

12. Rosenblat JD, Lee Y, McIntyre RS. The effect of pharmacogenomic testing on response and remission rates in the acute treatment of major depressive disorder: A meta-analysis. Vol. 241, Journal of Affective Disorders. Elsevier B.V.; 2018. p. 484–91.

13. Zeier Z, Carpenter LL, Kalin NH, Rodriguez CI, McDonald WM, Widge AS, et al. Clinical implementation of pharmacogenetic decision support tools for antidepressant drug prescribing. Am J Psychiatry [Internet]. 2018 Sep 1 [cited 2021 May 17];175(9):873–86. Available from: www.pharmgkb.org

14. Hiemke C, Bergemann N, Clement HW, Conca A, Deckert J, Domschke K, et al. Consensus Guidelines for Therapeutic Drug Monitoring in Neuropsychopharmacology: Update 2017 [Internet]. Vol. 51, Pharmacopsychiatry. Georg Thieme Verlag; 2018 [cited 2021 May 7]. p. 9–62. Available from: https://pubmed.ncbi.nlm.nih.gov/28910830/
